# How to develop a more accurate risk prediction model when there are few events

**DOI:** 10.1136/bmj.h3868

**Published:** 2015-08-11

**Authors:** Menelaos Pavlou, Gareth Ambler, Shaun R Seaman, Oliver Guttmann, Perry Elliott, Michael King, Rumana Z Omar

**Affiliations:** 1 Department of Statistical Science, University College London, WC1E 6BT London, UK; 2 Medical Research Council Biostatistics Unit, Cambridge; 3 School of Life and Medical Sciences, Institute of Cardiovascular Science, University College London; 4 Inherited Cardiac Disease Unit, the Heart Hospital, London; 5 Division of Psychiatry, University College London

## Abstract

When the number of events is low relative to the number of predictors, standard regression could produce overﬁtted risk models that make inaccurate predictions. Use of penalised regression may improve the accuracy of risk prediction

**Summary points**

Risk prediction models are used in clinical decision making and are used to help patients make an informed choice about their treatment
Model overfitting could arise when the number of events is small compared with the number of predictors in the risk model
In an overfitted model, the probability of an event tends to be underestimated in low risk patients and overestimated in high risk patients
In datasets with few events, penalised regression methods can provide better predictions than standard regression

Risk prediction models that typically use a number of predictors based on patient characteristics to predict health outcomes are a cornerstone of modern clinical medicine.^1^ Models developed using data with few events compared with the number of predictors often underperform when applied to new patient cohorts.^2^ A key statistical reason for this is “model overfitting.” Overfitted models tend to underestimate the probability of an event in low risk patients and overestimate it in high risk patients, which could affect clinical decision making. In this paper, we discuss the potential of penalised regression methods to alleviate this problem and thus develop more accurate prediction models.

Statistical models are often used to predict the probability that an individual with a given set of risk factors will experience a health outcome, usually termed an “event.” These risk prediction models can help in clinical decision making and help patients make an informed choice regarding their treatment.^3^ ^4^ ^5^ ^6^ Risk models are developed using several risk factors typically based on patient characteristics that are thought to be associated with the health event of interest (box 1). These predictors are usually selected on the basis of clinical experience and following a literature review. Given patient characteristics, the risk model can calculate the probability of a patient having the event. However, before a risk model is used in clinical practice, the predictive ability of the model should be evaluated. This process is known as model validation*,* and involves an assessment of calibration (the agreement between the observed outcomes and predictions) and discrimination (the model’s ability to discriminate between low and high risk patients).^2^ Typically, the model is validated internally (for example, using bootstrapping^7^ in box 2) or externally using patient data not used for model development (box 1).

**Box 1: Development and validation of a risk model with a binary outcome**

**Development**

This stage is when information on a binary outcome and predictor variables in a patient cohort is obtained, and a risk model is constructed. For illustration, we consider here an example outcome and set of predictor variables.

- Outcome: mechanical failure of artificial heart valve (yes *v* no)
- Predictor variables: sex (score of 1=female), age (years), body surface area (BSA; m^2^), and whether a replacement valve came from a batch with fractures (score of 1=valve came from batch with fractures)

A risk model relates the risk of a patient experiencing an event to a set of predictors. A common choice is the logistic regression model, which takes the form:

- Patient’s risk of heart failure = exp(patient’s risk score) ÷ (1+exp(patient’s risk score))
- Where patient’s risk score = intercept + (b_sex_×sex) + (b_age_×age) + (b_BSA_×BSA) + (b_fracture_×fracture);
- And b_sex_, b_age_, b_BSA_, and b_fracture_ are regression coefficients that describe how a patient’s values of the predictor variables affect risk.

The regression coefficients are estimated as those values that optimise the ability of the model to predict the outcomes in the patient cohort. This is called “fitting the risk model,” and can be achieved using various methods, such as standard logistic regression, ridge, or lasso.

**Prediction**

To predict risk, the fitted risk model is used to calculate a risk score for each patient. For example, if the estimated regression coefficients are as follows:

- b_sex_ = −0.193
- b_age_ = −0.0497
- b_BSA_= 1.344
- b_fracture_ = 1.261
- Intercept = −4.25

The risk score for a 40 year old female patient with a body surface area of 1.7 m^2^ and an artificial valve from a batch with fractures would then be calculated as:

−4.25 + (−0.193×1 (female sex)) + (−0.0497×40 (age in years)) + (1.344×1.7 (BSA in m^2^)) + (1.261×1 (fracture present in batch)) = −2.89

Therefore, her predicted risk would be:

- exp(−2.89) ÷ (1+exp(−2.89)) = 5.3%

**External validation**

For external validation, a completely new cohort of patients with information on the same outcome and predictors is studied. The estimated regression coefficients (from the development phase) are used to predict the risks for patients in the new cohort. The agreement between the predicted risks and observed outcomes is assessed—that is, the model is validated by evaluating performance measures that assess, for example, calibration and discrimination.

**Box 2: Bootstrap validation**

Bootstrap validation may be used when no external cohort of patients is available. The aim is to estimate how good the performance of the prediction model developed on the development set (the original dataset) would be on a hypothetical set of new patients. A bootstrap dataset is an imitation of the original dataset and is constructed by the random sampling of patients “with replacement” (that is, a patient can be selected more than once) from the original dataset.

Typically, a large number of bootstrap datasets (for example, 200) is created. Each dataset acts as a development dataset. In the simplest form of internal validation for the performance measure of a calibration slope:

- The model is fitted to each bootstrap dataset
- The estimated coefficients are used to obtain predictions for the patients in the original dataset
- These predictions are used to calculate the calibration slope for the fitted model.

The 200 estimates (one estimate for each bootstrap dataset) of the calibration slope are then averaged. For other performance measures—for example, the area under the receiver operating characteristic (ROC) curve—optimism adjusted measures can be obtained using a similar procedure.

In practice, datasets used in risk model development often contain few events compared with the number of candidate predictors, particularly when the event of interest is rare. An example would be structural failure of mechanical heart valves^8^ and sudden cardiac death in patients with hypertrophic cardiomyopathy.^6^ In such situations, use of standard regression methods to develop risk models could accurately predict outcomes for patients in the dataset used to develop the model, but may often perform less well in a new patient group. This difference is because the fitted model captures not only the underlying clinical associations between the outcome and predictors, but also the random variation (noise) present in the development dataset. This problem is called “model overfitting.” An overfitted model typically underestimates the probability of an event in low risk patients and overestimates it in high risk patients.^2^ This is known as poor calibration and has important consequences for clinical decision making. For example, overestimation of sudden cardiac death risk could lead to the unnecessary recommendation of implantable cardioverter defibrillators, exposing patients to surgical complications and wasting resources.^6^

This article focuses on ridge and lasso, two popular regression methods that can be used to alleviate the problem of model overfitting and are recommended in the TRIPOD checklist for developing and validating prediction models.^9^ Their ability to provide more accurate predictions than standard methods when there are few events is illustrated in two clinical examples.

## Sample size calculation for developing risk prediction models

When developing a risk model, a rule of thumb based on the events per variable (EPV) ratio is often used to determine the sample size. The EPV is the number of events in the data divided by the number of regression coefficients in the risk model. (Note that if variable selection is performed, the number of regression coefficients refers to the initial set of predictors, before variable selection.) It has been suggested that an EPV of 10 or more is needed to avoid the problem of overﬁtting.^7^ ^10^ For example, a dataset should contain at least 60 events to fit a risk model with six regression coefficients. When the EPV is smaller than 10, the effect of overfitting is pronounced.^11^

The development of a risk model often begins with a systematic review of the literature and consultation with clinical experts to identify a set of candidate predictors. However, even when this procedure is followed, an EPV of 10 may be difficult to achieve in studies involving few events, and therefore researchers often consider ways to reduce the number of predictors before developing the model. 

There are two common strategies. The first is univariable screening, where each predictor’s relation with the outcome is examined individually and only statistically significant predictors are included in the risk model. The second strategy is stepwise model selection (for example, backwards elimination), where predictors that are not statistically significant at a prespecified P value are removed in a stepwise manner from a model that initially includes all candidate predictors. However, both approaches have serious drawbacks—for example, the predictor selection process may not be stable (small changes in the data or in the predictor selection process could lead to different predictors being included in the final model).^7^ ^11^ ^12^ ^13^

## Shrinkage methods

Another way to alleviate the problem of model overﬁtting is to use methods that tend to shrink the regression coefficients (towards zero). Shrinking the regression coefficients has the effect of moving poorly calibrated predicted risks towards the average risk, and could assist in making more accurate predictions when the model is applied in new patients.^11^ ^14^

The simplest method is to shrink the regression coefficients by a common factor—for example, 20%—after they have been estimated by standard regression*.* This factor can be chosen using bootstrapping.^7^ ^15^ However, this approach does not perform well if the EPV is very low,^14^ and we do not discuss it further. An alternative approach, which is the focus of this paper, is to incorporate shrinkage as part of the model fitting procedure.

### Penalised regression

Penalised regression is a flexible shrinkage approach that is effective when the EPV is low (<10). It aims to fit the same statistical model as standard regression but uses a different estimation procedure.

The process of fitting a penalised regression model is as follows. Firstly, the form of the risk model (for example, logistic or Cox regression for binary and survival data, respectively) is specified using all candidate predictors. Next, the model is fitted to the data to estimate the regression coefficients. In standard logistic or Cox regression, the coefficients are estimated without imposing any constraints on their values. In datasets with few events, the range of the predicted risks is too wide as result of overfitting, but this range can be reduced by shrinking the regression coefficients towards zero. Penalised regression achieves this by placing a constraint on the values of the regression coefficients. The penalised regression coefficient estimates are typically smaller than those from standard regression. Several penalised methods that use different constraints have been proposed.^13^ ^16^ ^17^ We focus on ridge and lasso,^14^ arguably the two most popular shrinkage methods.

#### Ridge regression

Ridge fits the risk model under the constraint that the sum of the squared regression coeﬃcients does not exceed a particular threshold.^17^ ^18^ The threshold is chosen to maximise the model’s predictive ability, using cross validation*.* In cross validation, the dataset is split into *k* groups. The model is fitted to the (*k*−1) groups and validated on the omitted group. This procedure is repeated *k* times, each time omitting a different group.

#### Lasso regression

Lasso is similar to ridge, but constrains the sum of the absolute values of the regression coefficients.^16^ Unlike ridge, lasso can effectively exclude predictors from the final model by shrinking their coeﬃcients to exactly zero. Both ridge and lasso regression are readily available in software such as *R *(for example, package “penalized”) and SPSS.

In health research, where a set of prespecified predictors is often available, ridge regression is usually the preferred option.^14^ However, lasso might be preferred if a simpler model with fewer predictors (without affecting the predictive ability of the model) is desired, for example, to save time or resources by collecting less information on patients.

## How to detect model overfitting

An overfitted model could be detected through an assessment of model calibration using either an internal validation technique or external validation.^7^ This may be done by dividing the patients into risk groups according to their predicted risk, and comparing the proportion of patients who experienced the event in each group with the average predicted risk in that group, using a graph (calibration plot^2^) or table (which leads to the Hosmer-Lemeshow test^19^). 

Alternatively, the degree of overﬁtting may be quantified using a simple regression model. For binary outcomes, the outcomes in the validation data are regressed using logistic regression on their predicted risk scores (box 1). If the model is well calibrated, the estimated slope (or calibration slope) should be close to 1, whereas an overﬁtted model would have a slope much less than 1, indicating that low risks are underestimated and high risks are overestimated.^2^

## Application of penalised regression

The use of ridge and lasso methods can be illustrated by using data for 3118 patients with mechanical heart valves.^8^ The event of interest was the mechanical failure of the artificial valve, which occurred in only 56 individuals. The candidate predictors in this analysis were patient age, sex, BSA, fractures in the batch of the valve (no *v* yes), year of valve manufacture (before 1981 *v* after 1981), and valve size or position modelled using six clinically meaningful combinations constructed according to their expected levels of risk. A logistic regression model was used for illustrative purposes, with 10 coefficients. The EPV is 56/10=5.6, well below the recommended minimum of 10.

Standard, ridge, and lasso regression were used to estimate the regression coefficients shown in the table[Table tbl1]. We also used backwards elimination (with a 15% significance level^14^), which excluded the variable sex from the model (coefficients not shown).

**Table tbl1:** Estimates of regression coefficients, calculated by standard regression and penalised methods

Predictors	Descriptive statistics†	Regression coefficient estimates*		
		Standard regression	Ridge regression	Lasso regression
Intercept	—	−7.80	−5.97 (23)	−6.65 (15)
Sex (female)	1337 (43)	−0.24	−0.14 (41)	−0.16 (34)
Age (years)	54.1 (10.8)	−0.052	−0.047 (11)	−0.050 (4)
Body surface area (m^2^)	1.6 (0.3)	1.98	1.52 (24)	1.75 (12)
Aortic size 23, 27, 29, 31 mm	692 (22)	1.43	0.36 (75)	0.61 (68)
Mitral size 23-27 mm	369 (12)	1.30	0.22 (84)	0.43 (67)
Mitral size 29 mm	611(20)	1.95	0.80 (59)	1.13 (42)
Mitral size 31 mm	656 (21)	2.62	1.38 (47)	1.77 (33)
Mitral size 33 mm	104 (3)	2.58	1.41 (45)	1.73 (33)
Fracture in batch (yes)	1108 (35)	0.59	0.69 (−17)	0.64 (−9)
Date of manufacture (after 1981)	2363 (76)	1.38	1.02 (26)	1.22 (12)

*For ridge and lasso methods, numbers in brackets are percentages and represent the shrinkage compared with the standard regression estimates.

†For descriptive statistics, data are mean (standard deviation) for continuous predictors (age and body surface area), and number (percentages) for binary predictors.

The ridge and lasso coefficients were reduced compared with those from the standard regression model, with the greatest shrinkage applied to the valve size and position predictors (45-84% shrinkage for ridge and 33-68% for lasso). The shrinkage is reflected by the predicted risks, especially for high risk patients. Consider, for example, a female patient aged 20.5 years and with 1.7 m^2^ BSA, who had a 31 mm mitral valve manufactured after 1981 from a batch without fractured implants. Using the estimated coefficients from standard regression (table), the risk score for this patient is calculated by the following formula:

Risk score = −7.8 (intercept) + (−0.24×1(female sex)) + (−0.052×20.5(age; years)) + (1.98×1.7(BSA; m^2^)) + (2.62×1(mitral size 31 mm)) + (0.589×0(no fracture)) + (1.38×1(date of manufacture after 1981)) = −1.714.

Therefore, the predicted risk of mechanical failure is:

exp(−1.714) ÷ (1+exp(−1.714)) = 18% (average risk is 1.8%).

When the estimated coefficients from ridge and lasso are used instead, the predicted risks are less extreme: 12% and 15%, respectively. Figure 1 confirms that there are fewer extreme risk scores after applying shrinkage.

**Figure fig1:**
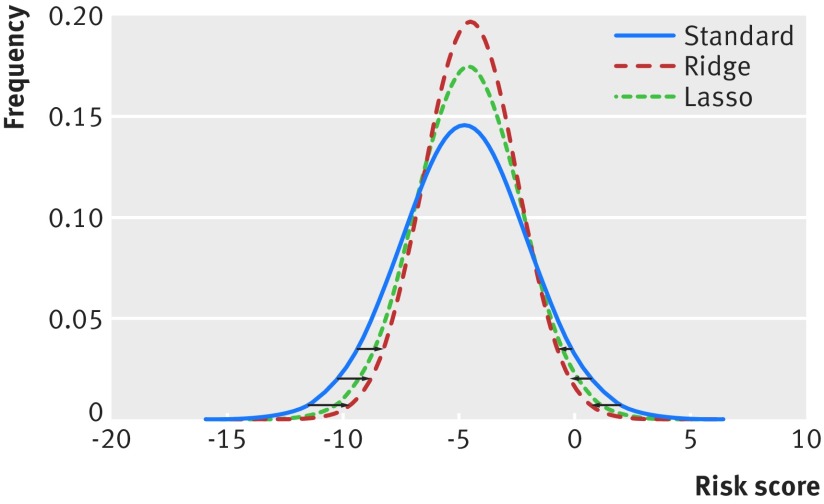
**Fig 1** Distribution of predicted risk scores estimated using standard, ridge, and lasso regression

The predictive performances of the risk models (developed using standard regression, backwards elimination, ridge, and lasso) were assessed using bootstrap validation (box 2).^7^ Calibration was assessed using the calibration slope and a calibration plot. Discrimination was measured by the commonly used area under the ROC curve measure, where a value of 1 suggests perfect discrimination and a value of 0.5 suggests no discrimination.

Standard regression produced an overfitted model (calibration slope 0.76 (95% confidence interval 0.65 to 0.99)), whereas the models from ridge and lasso demonstrated far better calibration (calibration slopes of 1.01 and 0.94, respectively). The calibration plot in figure 2 shows the observed proportion of patients who experienced the event and the average of their predicted risks in each of the four groups. Clearly, the standard risk model severely overestimates the risk of valve fracture for patients at the highest risk, which in practice might lead to patients undergoing unnecessary valve explant surgery. All three risk models (from standard, ridge, and lasso regression) demonstrated similar discrimination (all ROC areas 0.80 (95% confidence interval 0.78 to 0.82)). Backwards elimination also produced an overfitted model (calibration slope 0.77) with similar discrimination (ROC area 0.795). A second example illustrates the external validation of risk models (based on Cox regression) for sudden cardiac death in patients with hypertrophic cardiomyopathy (web appendix).

**Figure fig2:**
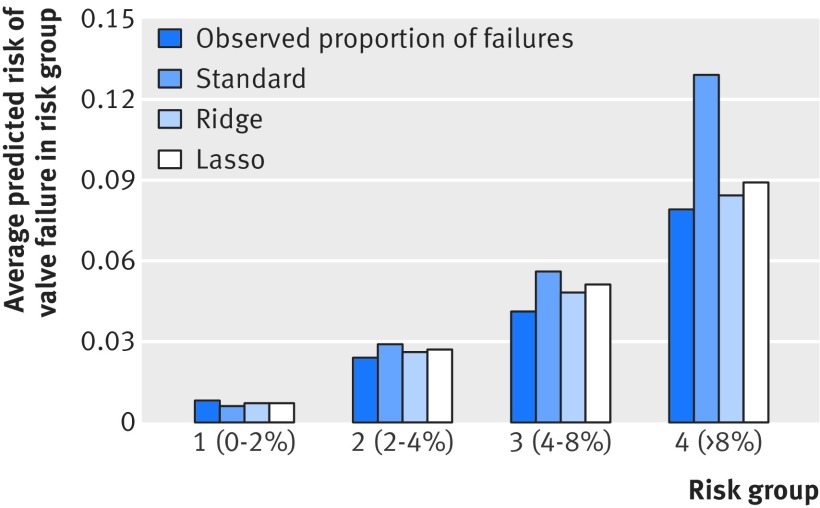
**Fig 2** Observed proportions versus average predicted risk of the event (using standard, ridge and lasso regression). Overestimation of risk for high risk patients can be seen when standard regression is used

## Conclusion

When the number of events is low relative to the number of predictors in the risk model, standard regression may produce overﬁtted risk models that make inaccurate predictions. Common approaches to reduce the number of predictors in a risk model, such as stepwise selection or univariable screening, are problematic and should be avoided.^7^ ^14^ Often the EPV can still be small (<10) even after existing knowledge has been used to eliminate some of the initial candidate predictors. In such cases, it is recommended that the use of penalised regression methods be explored. Risk models produced using penalised regression generally show improved calibration, and could also show improved discrimination.^14^

Other methods could be more appropriate in some situations.^13^ Notably, there may be scenarios where existing evidence (from published risk models, meta-analysis, and expert opinion) can be incorporated in the estimation procedure. These contributions could lead to better predictions than those obtained from ridge and lasso.^20^ ^21^ In this paper, we focused on the issue of model overfitting, but small datasets and datasets with few events are also susceptible to other problems, especially when binary predictors with a very low (or high) prevalence are present; in such scenarios other methods may be more suitable than ridge and lasso.^22^

## Notes

Ethical approval: Not required.

Data sharing: It is not possible to make the original heart valve replacement data available owing to confidentiality issues. The data are only available for methodological research by the lead investigators who were involved in the actual clinical research of these heart valves. RO was one of the lead investigators for this research project, which was carried out 11 years ago.

Transparency: MP affirms that the manuscript is an honest, accurate, and transparent account of the study being reported; that no important aspects of the study have been omitted; and that any discrepancies from the study as planned (and, if relevant, registered) have been explained.

## Notes

Cite this as: *BMJ* 2015;351:h3868
