# Supplementary material for: How to develop a more accurate risk prediction model when there are few events
Source: BMJ. 2015 Aug 11;351:h3868. doi: 10.1136/bmj.h3868 (PMC4531311; doi:10.1136/bmj.h3868)
Supplement: Supplementary file 1 [file pavm022798.ww1_default.pdf]

Supplementary material for the article 'How to develop a more accurate risk prediction model when there are few events'

Menelaos Pavlou *research associate*[1], Gareth Ambler *senior lecturer*[1], Shaun R Seaman *senior statistician*[2], Oliver Guttmann[3] *cardiology registrar*, Perry Elliott *professor*[4], Michael King *professor*[5], Rumana Z Omar *professor*[1]

Application: Example with external validation

A risk model was proposed[1] to predict the risk of sudden cardiac death (SCD) in Hypertrophic cardiomyopathy (HCM) patients within 10 years from diagnosis. Patients at high predicted risk of SCD are candidates for implantation of Implantable Cardioverter Defibrilators (ICD) which regulate cardiac arrhythmias and can reduce the chance of SCD.

There were 11 pre-specified candidate predictors: age, maximal left ventricular wall thickness, fractional shortening, left atrial diameter, peak left ventricular outflow tract gradient (all continuous) and gender, family history of SCD, non-sustained ventricular tachycardia, severity of heart failure defined by NYHA class III/IV, unexplained syncope (all binary). A Cox regression model was used.

In this illustration of ridge and lasso methods, risk models were developed using data on 1000 patients from one centre (42 events). There were 11 regression coefficients and the EPV was 4.2. The models were externally validated using data from different centres (2405 patients, 106 events).

The coefficient estimates from standard, ridge and lasso regression are shown on Table 1S. As anticipated, standard regression yielded an overfitted model (calibration slope=0.79; 95% CI= (0.56-0.99)), compared to the well-calibrated ridge and lasso models (calibration slope=1.05; 95% CI (0.78, 1.35) and 1.02; 95% CI= (0.74-1.30), respectively). The lasso method excluded three predictors (fractional shortening, sex and severity of hypertrophy), whilst retaining a good predictive performance. All methods had identical discrimination (as measured by the C-index,[2] a discrimination measure for survival data analogous to area under the ROC curve): C-index=0.732 (95% CI=(0.720-0.745)) . The calibration plot in Figure 1S shows the observed proportion of patients with the event and the average of their predicted risks in each of the four risk groups, demonstrating that the standard risk model overestimates the risk sudden cardiac death in the highest-risk patients.

	Standard	Ridge	Lasso
age (years)	-0.024	-0.015	-0.015
mwt (mm)	0.043	0.038	0.039
fs (mm)	0.002	0.003	0
la diameter (mm)	0.042	0.028	0.027
peak lvotg (mmHg)	0.009	0.007	0.007
scd family	0.60	0.43	0.42
nsvt	0.30	0.19	0.03
syncope	0.93	0.71	0.74
sex-male	-0.14	-0.07	0
NYHA class III/IV	-0.24	-0.07	0

Table 1S: Regression coefficient estimates using standard, ridge and lasso regression. Lasso excluded three predictors by shrinking their corresponding coefficients to exactly zero. Abbreviations: mwt: maximal wall thickness; la diameter: left atrium diameter; fs: fractional shortening; lvotg: left ventricular outflow tract gradient; nsvt: non-sustained ventricular tachycardia; NYHA III/IV: New York Heart Association Class III/IV.

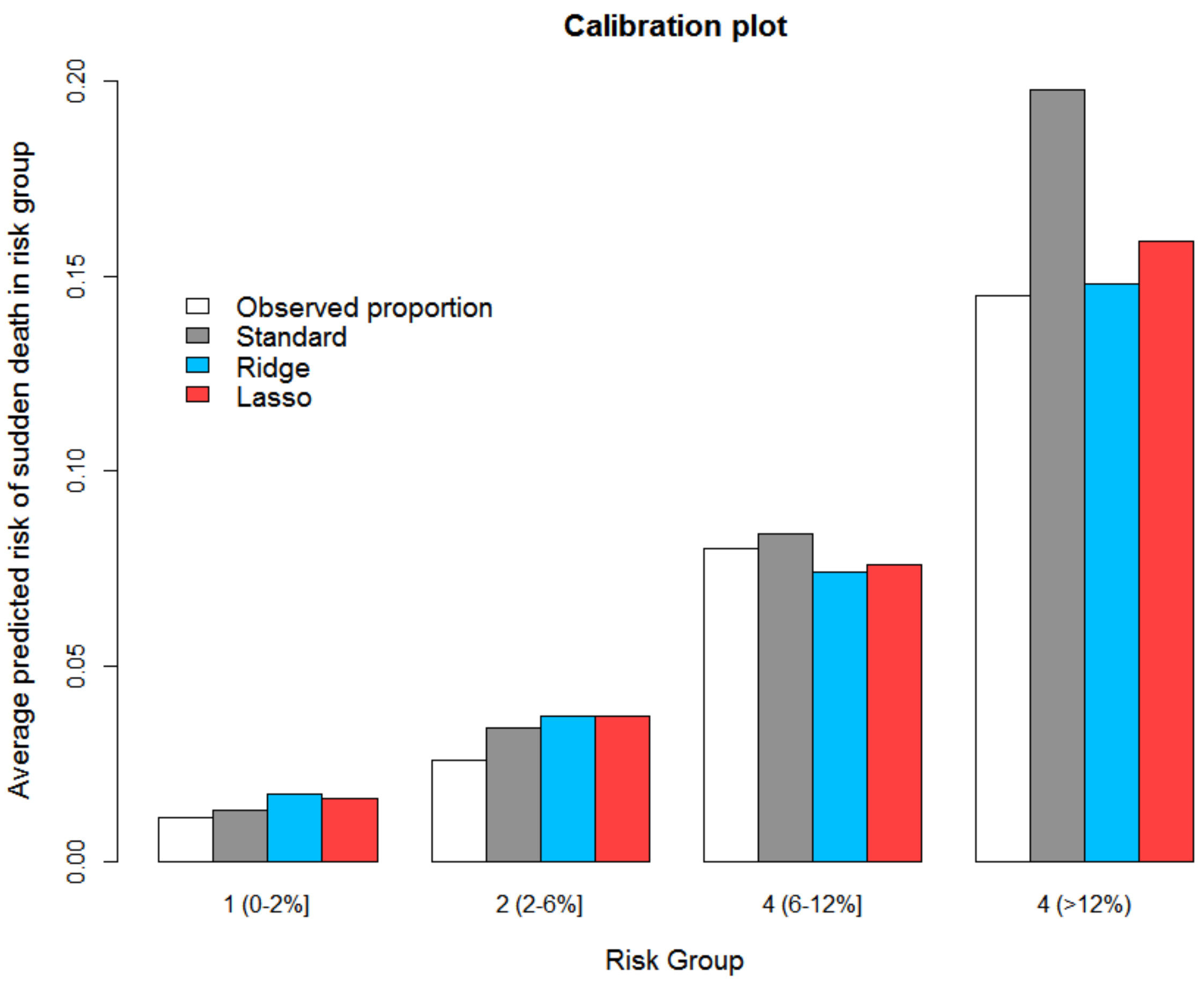

Figure 1S: Observed proportions versus average predicted risk of sudden death (using standard, ridge and lasso regression), demonstrating over-estimation of risk for the high-risk group when standard regression is used.

References

1 O'Mahony C, Jichi F, Pavlou M, et al. A novel clinical risk prediction model for sudden cardiac death in hypertrophic cardiomyopathy. *European heart journal* 2013.

2 Uno H, Cai T, Pencina MJ, D'Agostino RB, Wei LJ. On the C-statistics for Evaluating Overall Adequacy of Risk Prediction Procedures with Censored Survival Data. *Statistics in medicine* 2011;**30**(10):1105-17.
